# Advances of Traditional Chinese Medicine Regulating Connexin43 in the Prevention and Treatment of Myocardial Infarction

**DOI:** 10.1155/2021/8583285

**Published:** 2021-11-15

**Authors:** Keke Liu, Meng Lv, Xiaodi Ji, Lixia Lou, Bo Nie, Jiuli Zhao, Aiming Wu, Mingjing Zhao

**Affiliations:** Dongzhimen Hospital, Beijing University of Chinese Medicine, Key Laboratory of Chinese Internal Medicine of Ministry of Education and Beijing, Beijing 100700, China

## Abstract

Gap junctions are the main form of interaction between cardiomyocytes, through which the electrochemical activities between cardiomyocytes can be synchronized to maintain the normal function of the heart. Connexins are the basis of gap junctions. Changes in the expression, structural changes (e.g., phosphorylation and dephosphorylation), and distribution of connexins can affect the normal electrophysiological activities of the heart. Myocardial infarction (MI) and concurrent arrhythmia, shock, or heart failure can endanger life. The structural and functional damage of connexin (Cx) 43 in cardiomyocytes is a central part of the pathological progression of MI and is one of the main pathological mechanisms of arrhythmia after MI. Therefore, increasing Cx43 expression has become one of the main measures to prevent MI. Also, intervention in Cx43 expression can improve the structural and electrical remodeling of the myocardium to improve MI prognosis. Here, research progress of Cx43 in MI and its prevention and treatment using Traditional Chinese Medicine formulations is reviewed.

## 1. Overview of Connexins (Cxs)

Cxs are transmembrane proteins that assemble to form gap junctions (GJs) in vertebrate animals. GJs are communication connections found widely in tissues except blood cells and skeletal-muscle cells. GJs are distributed in a patchwork pattern and are one of the main forms of cell connection. Six rod-shaped Cxs combine to form hydrophilic tubules of diameter ∼2 nm to constitute one hemichannel. The hemichannels on both sides of adjacent cell membranes are linked to each other to form a direct connection channel between cells. The exchange of ions and small molecular substances (e.g., amino acids, glucose, nucleotides, vitamins, hormones, growth factors, and cyclic adenosine monophosphate) has a role in transmitting chemical information, coordinating cell metabolism, and regulating the growth and differentiation of cells. In addition, the electrical resistance at a GJ is low, and the conduction speed is rapid and accurate, which ensures the synchronized contraction of tissues and organs. As dynamic structures, GJs not only vary in number but also open or close channels by sliding through each other. For example, changes in the membrane potential, pH, or calcium ion (Ca^2+^) concentration can change the state of channels.

**Figure 1:**
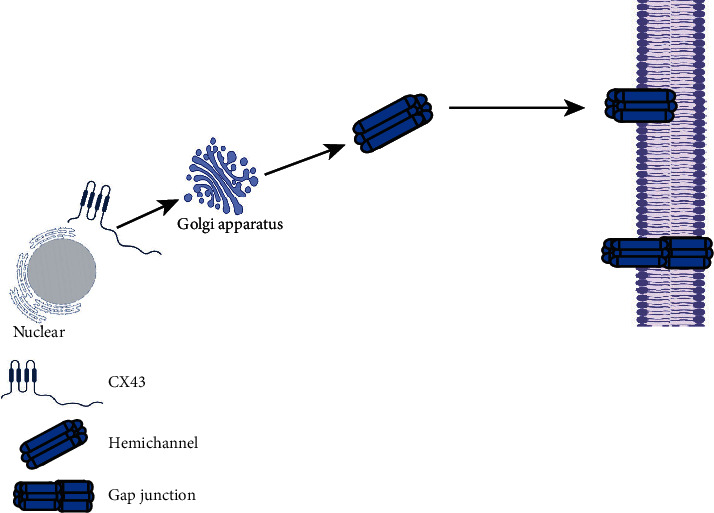
Structure of a gap junction.

Proteins are the structural basis of GJs. Abnormal expression of GJs leads to abnormal electrochemical coupling and cardiac dysfunction. In mammals, GJ proteins are a large class of membrane proteins encoded by a multigene family, including Cx26, Cx30, Cx31, Cx32, Cx33, Cx37, Cx40, Cx43, Cx45, Cx46, Cx50, and Cx57. Cxs have four transmembrane domains (M1–M4), two extracellular rings (E1 and E2), and three intracellular segments (N terminus (N-), C terminus (C-), and cytoplasmic ring). Among them, the variation in E1 and E2 and M3 is large, but not in C- and N- termini. These differences lead to polymorphism in the family. The phosphoric acid of Cx43 is generated mainly on sericin acid [1, 2]. Cxs can be divided into three categories according to the relative molecular weight of genes. Group I (*β* class) includes Cx26, Cx30, Cx31, and Cx32. Group II (*α*2) includes Cx33, Cx37, Cx40, Cx43, Cx45, Cx46, Cx50, and Cx57. Group III (type *γ*^2^) is mainly represented by Cx36, which was discovered recently [3]. Like other membrane proteins, the ribosome of a Cx on a rough endoplasmic reticulum (rER) undergoes preliminary translation and then transfers to the ER for processing. This action involves obtaining four transmembrane structures and the assembly of Cx32 proteins into hexamers and is followed by entry into the Golgi apparatus for processing and assembly. For example, the Cx43 protein is assembled into hexamers, which are transported by the Golgi apparatus to the cell membrane in microtubule-dependent or microtubule-independent manners, and open after docking with the Cx of adjacent cells (Figure 1). Clusters of GJs gather to form “junction plaques.” The latter are in dynamic equilibrium because they integrate new junctions continuously from the outer edge and internalize the center of the junction plaque into one side of the cytoplasm.

Changes in transcription, translation, modification, assembly, and transport of Cxs, as well as abnormal formation of connecting channels and connecting plaques, affect the quality and quantity of communication between cells, thereby affecting electrochemical coupling and leading to cardiac dysfunction. The main Cxs expressed in cardiomyocytes are Cx43, Cx40, Cx45, and Cx43.  Section 2 discusses the role of Cx43 specifically in myocardial infarction (MI).

## 2. Pathological Changes of Cx43 after MI

“Cx43 remodeling” refers to downregulation of expression of Cx43 protein, increased lateral distribution, and altered phosphorylation caused by MI. In normal hearts, Cx43 is present mainly in the phosphorylated form. MI [4, 5] leads to increased activity in sympathetic nerves and promotes the degradation and dephosphorylation of Cx43 [6]. Studies have shown that the contents of Cx43 protein, phosphorylated Cx43 protein, and Cx43 mRNA are decreased significantly after acute MI in dogs, and that ablation of MI can increase the contents of Cx43 protein, phosphorylated Cx43 protein, and Cx43 mRNA [7]. In addition to the downregulation and dephosphorylation of Cx43, a lateral distribution of connections increases the anisotropy of ventricular-muscle propagation and the number of semichannels formed by Cx43 and increases the outflow of adenosine triphosphate and sodium ion (Na+) inflow of cardiomyocytes, leading to delayed depolarization [8], which is likely to cause arrhythmias. In addition, Cx43 remodeling shows different manifestations in different pathological stages. For example, Xiaoxing and colleagues found that Cx43 expression increased during simulated ischemic preconditioning and speculated that Cx43 might be involved in this process [9].

**Figure 2:**
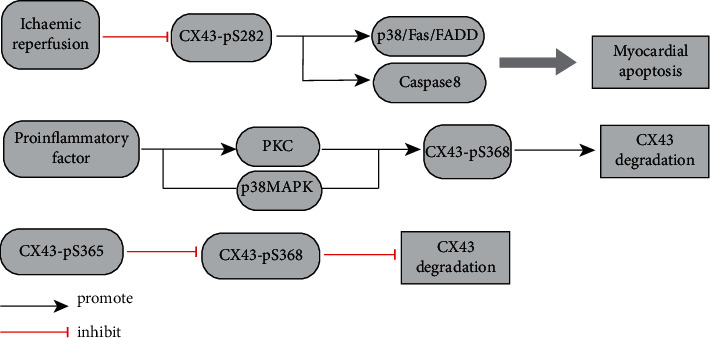
Pathological changes of Cx43 after myocardial infarction.

The changes in channel function mediated by the phosphorylation status of different phosphorylation sites of Cx43 are essential for GJ communication. Studies have shown that expression of Cx43-pS262 and Cx43-pS368 increases and expression of Cx43-pS282 decreases, in a rat heart with ischemia-reperfusion injury (30 min/120 min). Cx43-pS282 is involved in regulating the survival and electrical stability of cardiomyocytes. Reduced expression of Cx43-pS282 activates p38/Fas/FADD (Fas-associating protein with a novel death domain) and caspase-8 pathways, leading to increased apoptosis of cardiomyocytes [10] (Figure 2). In addition, Cx43-pS282 can regulate the phosphorylation of S279 in cardiomyocytes, and both are involved in the maintenance of cardiomyocyte homeostasis [11]. However, various proinflammatory factors and proteins activate the protein kinase C and p38 mitogen-activated protein kinase (MAPK) signaling pathways to increase the phosphorylation of Cx43 at position 368, inhibit the function of the Cx43 GJ channel, and trigger an abnormality of the QRS wave group [12]. The reason for this phenomenon is that direct phosphorylation of the S368 site of Cx43 leads to closure and degradation of the channel gate. In contrast to the S368 site of Cx43, cyclic adenosine monophosphate- and protein kinase A-dependent phosphorylation of the S365 site of Cx43 enhances the expression of GJs and opens the channel gate. Studies have also shown that phosphorylated-S365 may have a role because it prevents the phosphorylation of Cx43 at S368 [13, 14]. In addition, phosphorylation at S368 may have a role in key events in the cell cycle. Studies have shown that the phosphorylation level of S368 in the S phase and G2/M phase of the cell cycle increases significantly, whereas the number of functional connection channels and intercellular communication decrease, that is, the phosphorylation level at the S368 site is negatively correlated with GJ assembly [13].

## 3. Factors Influencing Cx43 Remodeling after MI

**Figure 3:**
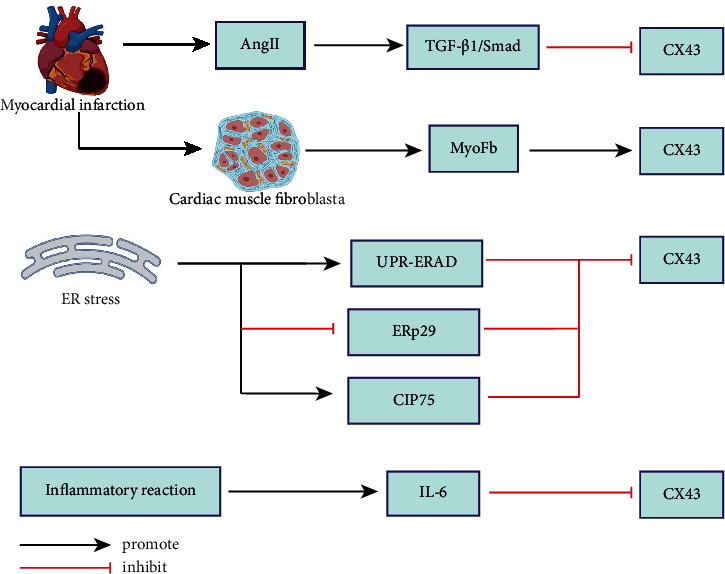
Factors influencing Cx43 remodeling after myocardial infarction.

Studies have shown that MI leads to activation of angiotensin II and the downregulation of Cx43 expression by acting on the transforming growth factor-beta 1/single mothers against decapentaplegic (TGF-*β*1/Smad) pathway [15]. Other studies have shown that endoplasmic reticulum stress (ERS) may be the key factor affecting the expression of GJ of Cx43. First, ERS and GJ dysfunction coexists in ischemic heart disease. They also have a similar effect on cellular behavior, such as promoting cell growth and increasing resistance to stress caused by hypoxia, drugs, or radiation. Second, the ER is required to participate in the formation and transport of GJ proteins to the surface of cell membranes through secretory pathways, and UPR (unfolded protein reaction) can downregulate expression of the genes encoding Cx to reduce the load on the ER. A considerable part of the newly synthesized junction molecules will also be degraded by ER-associated degradation. Studies have shown that the ER-related chaperone ERP29 is associated with stabilizing Cx43 monomers in the ER, and that interfering with ERP29 expression inhibits Cx43 secretion and reduces the efficiency of Cx43 for forming GJs [16]. CIP75 belongs to a family of proteins containing the UBL UBA domain, which mediates the interaction with Cx43. The interaction domain between Cx43 and CIP75 is located at multiple phosphorylation sites between Lys264 and Asn302 of the Py sequence (Figure 3). The UBL domain interacts with the S2/RPN1 and S5a/RPN10 protein subunits of the regulated 19S proteasome cap subunit of the 26S proteasome complex. Overexpression experiments have shown that CIP75 significantly increases the degradation of Cx43 and reduces its half-life, thereby participating in Cx43 turnover [17]. Some scholars have suggested that GJs control the fate of cells against stress. To maintain the homeostasis of tissues and organs, the coordinated response of cells to ERS involves regulating Cxs. Mesangial cells and ERS-inducing factors together lead to downregulation of Cx43 at protein and mRNA levels. This process and ERS significantly inhibit the activity of Cx43 gene promoters, reduce [35S]-methionine incorporation of Cx43 protein, and accelerate degradation of Cx43 protein [18]. A novel interactor of Cx43 in the heart, Eps15 homology domain protein-1, can interact with Cx43 through Eps15 to regulate its endocytic transport, thereby participating in the pathological remodeling of Cx43; reducing its expression, as well as phosphorylation and ubiquitination of Cx43, can promote this process [19]. Ca^2+^ overload at the edge of MI is also associated with the expression and dysfunction of GJs [20].

Inflammation interferes with Cx43 expression. Regardless of the specific etiology and organ localization, systemic inflammation rapidly causes atrial electrical remodeling by downregulating expression of cardiac Cxs through increased expression of interleukin (IL) 6. Although this process is short-lived, these changes can significantly increase the risk of atrial fibrillation and related complications during active inflammation [21]. Some studies have found that, after MI, the intermediate product of the phospholipid metabolism of lysophosphatidic acid in cell membranes causes arrhythmia by regulating inflammatory cells and reducing expression of Cx43 protein [22]. Therefore, downregulation of expression of Cx43 protein may also be related to the inflammatory response of cells.

In addition, MI can cause resident fibroblasts to differentiate into myofibroblasts. Subsequently, myofibroblasts establish contact with cardiomyocytes and show high expression of Cx43, which results in a shortened duration of action potential of cardiomyocytes and hyperpolarization of the resting membrane potential [23]. Studies have shown that Cx43 expression in myofibroblasts increases after MI. IL-1*β* can increase the expression of Cx43 in myofibroblasts and reduce expression of its markers, thereby changing the cell phenotype and the interaction of cardiomyocytes to reduce the duration of contraction [24]. In addition, exogenous administration of TGF-*β* to cultures of normal heart myofibroblasts has been shown to result in the upregulation of Cx43 expression. One can speculate that the communication between the myofibroblasts in the infarct area and the myofibroblasts of the peri-infarct area increases and activates the TGF-*β* pathway and upregulates Cx43 expression [25].

Hence, one can postulate that the biochemical mediators that limit ischemic injury are transmitted between cells, and that cardiomyocytes downregulate Cx43 expression. Unlike cardiomyocytes, fibroblasts can upregulate Cx43 expression to maintain electrical and metabolic coupling if intercellular communication is impaired. Cx43 could be a potential target for preventing abnormal coupling of cardiomyocytes and proliferation of myofibroblasts at the border of the infarct area.

## 4. Targeted Cx43 Expression after MI

Cx43 can be used as a candidate indicator for the diagnosis of early myocardial ischemia (within the first 4–6 h after partial myocardial blood flow is interrupted) [26]. In patients with ischemic heart disease, the surface area of the GJ per unit cell volume is reduced by 47%, and abnormal cell coupling causes arrhythmia [27]. The increase in Cx43 expression has a positive effect on the recovery of damaged heart function and can reduce the occurrence of arrhythmias during regeneration of heart tissue [28, 29].

Targeting Cx43 expression can improve MI prognosis. For example, dexmedetomidine increases the distribution of Cx43 through phosphorylated-adenosine monophosphate-activated protein kinase, inhibits activation of nuclear factor-kappa B, and reduces spontaneous ventricular arrhythmias in ischemic heart disease caused by MI [30]. Verapamil and doxycycline can also target Cx43 to improve GJ coupling [31, 32]. Activation of M3 muscarinic acetylcholine receptors has a short-term protective effect on a heart with ischemic injury, the mechanism of which is related to the upregulation of cyclo-oxygenase-2 expression and inhibition of ischemic dephosphorylation of Cx43 [33]. Some tissue factors can increase Cx43 expression. Studies have shown that hepatocyte growth factor and insulin-like growth factor-1 can increase Cx43 expression in rats with MI, and insulin-like growth factor-1 may have a role through the MAPK/*p*38 and extracellular signal-regulated kinase (ERK)1/2 pathways [34]. The adrenomedullin secreted by the epicardium can drive the repair of cardiac lymphangiogenesis through Cx43 to improve myocardial edema after injury [31]. Integrin-linked kinase can also inhibit Cx43 remodeling by activating protein kinase B, reducing ventricular remodeling, and improving heart function [35].

“Gold nanopatches” have been used to treat MI. They can increase Cx43 expression in animals suffering from MI, increase blood-vessel density, and reduce the scar area [36]. In the ischemic phase of MI or at the beginning of reperfusion, intravenous administration of an inhibitor of p38 MAPK, SB203580, can reduce the occurrence of ventricular tachycardia/ventricular fibrillation, which is related to increasing the phosphorylation level of Cx43 [37]. Studies have found that mRNA expression of the Cx43 gene GJA1 can undergo other translations to generate smaller subtypes in the heart. GJA1-20k is the most abundant; it is an endogenous stress-response protein that can induce mitochondrial biogenesis and metabolic hibernation to protect the heart from ischemia-reperfusion injury [38]. The new multichannel blocker CPUY102122 can increase Cx43 expression in the left ventricle and reduce expression of np-43 significantly, thereby having antiarrhythmic and cardiovascular-protection effects [39]. Irradiation using carbon ions can also increase Cx43 expression, improve conductivity, and reduce the spatial heterogeneity of repolarization [40].

## 5. Traditional Chinese Medicine (TCM) Formulations Used to Regulate Cx43 Expression

Several clinical and experimental studies have demonstrated the efficacy of TCM formulations in MI treatment. Recent studies have shown that regulation of Cx43 expression to improve heart function is a potential target for several TCM formulations. For example, colleagues showed that a 2 : 1 combination of astragaloside IV and tanshinone IIA significantly increased the protein and mRNA expressions of Cx37, Cx40, and Cx43, modulated GJ communication, and promoted endothelial-cell proliferation and formation of tubular structures [41].

The change of Cx43 expression is an important part of Cx43 reconstruction, and some TCM formulations have a protective role by increasing Cx43 expression. For example, oxymatrine can reduce the incidence of myocardial injury and ventricular arrhythmia in MI rats, and the mechanism may be related to upregulation of Cx43 expression [42]. Tanshinone IIA (a fat-soluble pharmacologically active ingredient of danshen) can reduce arrhythmia risk after MI. It has a protective effect against myocardial ischemia after MI, and its mechanism of action is related to upregulation of Cx43 expression [43]. Tanshinone IIA and astragaloside IV can promote angiogenesis by upregulating Cx43 expression, which provides experimental evidence for their clinical application as treatment for coronary heart diseases [44]. Linalool has been shown to dose-dependently reduce the incidence of arrhythmias in a rat model of MI. We propose that the key mechanism behind this antiarrhythmic effect is probably the prevention of reduced Cx43 expression following MI [45]. Wu et al. demonstrated that the regulatory effects on Cx43 might be a possible mechanism of action of Wenxin granules in MI treatment at the gene level [46]. Tiaogan Qingxin granules have been shown to reduce the size of the myocardial infarct, lower the risk of MI, and increase Cx43 expression, possibly by increasing blood supply to cardiac muscles. Hence, Tiaogan Qingxin granules may be useful for treating ischemic PVB (premature ventricular beat) [47]. Fumai granules with ginseng as the main drug can significantly reduce the occurrence and duration of atrial fibrillation, which is related to increased expression of Cx43 and Cx40 [48]. Shexiang Baoxin pills can improve the cardiac function of rats with heart failure and upregulate the expression of GJ protein Cx43 in myocardial tissue [49]. Gegenqinlian decoction can improve the pathological changes and enzyme expression in the rat myocardium by increasing the Cx43 expression in the myocardium, so as to reduce the occurrence of ventricular fibrillation and tachycardia [50]. Shensong Yangxin capsules can improve cardiac dysfunction and arrhythmia after MI in rabbits, and its mechanism is related to increasing expression of Cx43 [51].

TCM formulations can not only regulate Cx43 expression directly but also regulate its spatial distribution, stability, and phosphorylation level. For example, Wenxin granules [52] and Sensen capsules (*Artemisia annua* and *Changshan annulata*) can improve the expression and distribution of Cx43 during myocardial ischemia, thereby reducing the occurrence of ischemic arrhythmias [53]. Aconitum Chishi Fang can increase the expression of CX43 protein and make it evenly distributed so as to improve myocarditis in rats with acute MI [54]. Compound Huangqi Yangxin mixture has a protective effect against ischemic arrhythmia, and its antiarrhythmic effect may be related to regulation of the structural stability of Cx43 [55]. The protective effect of Fumai decoction may be related to reducing the phosphorylation level of Cx43 Ser279/282 and regulating the phosphorylation of ERK1/2 and Cx43Ser262 [56].

The specific mechanism of the regulation of Cx43 by TCM formulations involves direct regulation of Cx43 expression, interfering with the electrochemical communication between cells and improving arrhythmia so as to prevent and treat MI. Furthermore, in theory, abnormalities in Cx43 could involve metabolism, signal transduction, and transportation. Hence, it is inherently difficult to distinguish between the exact causes of the structural and functional disorders associated with MI, which are interrelated. Therefore, TCM formulations may have a role by regulating related pathways. Studies have shown that Shensong Yangxin capsules can upregulate Cx43 expression and inhibit Ca^2+^ overload [57, 58]. Lianxia granules can increase the threshold of ventricular fibrillation in rats with MI, and the mechanism of action may be related to a reduction in the inflammatory response and improvement of Cx43 remodeling [59]. Shengmai injection and Xuesaitong injection can increase the threshold of ventricular fibrillation in rats with MI, and the mechanism of action is related to improving cardiac structure and Cx43 expression after MI [60]. Tongguan capsules and Dan Qi soft capsules can increase Cx43 expression, intervene in interstitial fibrosis and cardiomyocyte hypertrophy, reduce left-ventricular remodeling and dysfunction, and reduce the occurrence of arrhythmia [61, 62]. Cx43 is related to different biological processes, so the prescription for enriching qi and activating blood may also regulate the concentration of free Ca^2+^ in cardiomyocytes, improve the GJ communication between cells, repair the cytoskeleton and ultrastructure of cardiomyocytes, and thus, upregulate the expression of Cx43 protein and protect cardiomyocytes. The effects of chemical agents and TCM formulations on Cx43 expression are shown in Table 1.

TCM formulations may improve the changes of Cx43 expression related to MI through different ways and change the interaction between myocardial cells. In this way, TCM formulations may have unique advantages in the prevention and treatment of MI.

## 6. Summary

Acute MI is an acute and persistent myocardial necrosis caused by ischemia and hypoxia of coronary arteries that can be complicated with arrhythmia, shock, or heart failure and can often endanger life. Abnormal expression of Cx43 genes in myocardial cells and defects in communication of intercellular junctions are closely related to the occurrence and development of MI. Furthermore, a state change occurs in several sites of Cx43, which affects the gate characteristics, the permeability of the gap connection, and Cx43 assembly, and may have a role in specific pathological processes. MI often results in reduced Cx43 expression, increased lateral distribution, and altered phosphorylation status, whereas Cx43 regulates channel activity through phosphorylation/dephosphorylation and nitroso modification. Restoration and enhancement of Cx43 transcriptional and posttranscriptional levels can improve the electrochemical communication between cardiomyocytes as well as between cardiomyocytes and interstitial cells by enhancing communication between intercellular junctions, which is very important for MI treatment. Drugs targeting Cx43 interfere with intercellular electrochemical communication by increasing GJ expression. Studies have shown that the mechanism of action of TCM formulations in the prevention and treatment of cardiovascular diseases is related to the intervention of Cx43 expression. Therefore, further exploration of how they regulate Cx43 expression and investigation of other potential drug candidates will be worthwhile.

Cx43 can be used as a new target for the prevention and treatment of MI not only from the perspective of intercellular electrochemical communication and overall regulation of arrhythmias but also from the perspective of regulating various biological processes interacting with Cx43. Therefore, increasing Cx43 expression has become one of the main measures for the prevention and treatment of MI. The multitarget effect between different components and compatibility of TCM formulations will contribute to the effective prevention and treatment of MI.

## Figures and Tables

**Table 1:** Effects of drugs on CX43 expression.

Name	Targets	CX43 expression	References
Dex	AMPK,NF-*κ*b	CX43↑	[30]
Verapamil doxycycline	CX43	CX43↓	[31, 32]
M(3)-MACHRs	COX2	pCX43↓	[33]
HGF IGF-1	MAPK/*p*38,ERK1/2	CX43↑	[34]
ILK	AKT	CX43 remodeing↓	[35]
Gold nanopatch	CX43	CX43↑	[36]
CY22	CX43,np-CX43	CX43↑, np-CX43↓	[39]
Tanshinone IIA	CX43 mRNA	CX43↑	[43]
Linalool	CX43	CX43↑	[45]
Wenxinkeli	CX43	CX43↑	[46]
Fumai decoction	CX43 ser279/282 ERK1/2, CX43 ser262	CX43↑	[48]

## Data Availability

The data used to support the findings of this study are available from the corresponding author upon request.
